# Omics-derived signatures in metabolic dysfunction-associated steatotic liver disease: from molecular characterization to clinical translation

**DOI:** 10.3389/fmed.2026.1946657

**Published:** 2026-10-14

**Authors:** Ziyang Qin, Dulguun Juramt, Yuguo Chen, Yunyi Li, Jiaqi Wei, Hongyu Yang, Lanlan Chen, Wei Qiu

**Affiliations:** 1 Department of Hepatobiliary and Pancreatic Surgery, General Surgery Center, the First Hospital of Jilin University, Changchun, Jilin, China; 2 Department of Hepatology and Gastroenterology, Charité-Universitätsmedizin Berlin, Campus Virchow-Klinikum (CVK) and Campus Charité Mitte (CCM), Berlin, Germany

**Keywords:** biomarkers, metabolic dysfunction-associated steatohepatitis, metabolic dysfunction-associated steatotic liver disease, metabolomics, multi-omics, precision medicine, proteomics, spatial transcriptomics

## Abstract

Metabolic dysfunction-associated steatotic liver disease (MASLD) is a highly prevalent systemic metabolic disorder with heterogeneous clinical trajectories. Although liver biopsy remains the histological reference standard, its invasiveness and limited scalability restrict routine use, while established non-invasive tests mainly capture fibrosis-related structural changes and provide limited insight into molecular heterogeneity, active steatohepatitis and early fibrotic transition. Multi-omics technologies offer a mechanistically informed framework for resolving this heterogeneity. In this Review, we summarize recent advances in genomic, transcriptomic, single-cell and spatial, proteomic, lipidomic and metabolomic signatures and candidate biomarkers in MASLD. Genomic studies define inherited susceptibility and risk enrichment, whereas transcriptomic and spatial approaches reveal disease-associated pathways, cell states and tissue niches. Proteomics provides blood-accessible signals of steatohepatitis activity, matrix remodelling and systemic metabolic risk, while lipidomics and metabolomics capture lipotoxic lipid composition, mitochondrial stress, bile acid dysregulation and gut–liver metabolic perturbation. We further discuss how integrative multi-omics, systems biology and artificial intelligence can organize high-dimensional molecular data into disease modules, molecular subtypes, biomarker signatures and candidate therapeutic nodes. In addition, omics-informed studies of bioactive compounds and precision nutrition illustrate how nutritional modulation may be evaluated through molecular response profiles. Despite promising progress, most omics-derived biomarkers remain limited by cohort specificity, platform dependence, incomplete external validation and unclear clinical thresholds. Future translation will require longitudinal, spatially resolved and clinically anchored integration to support risk stratification, at-risk metabolic dysfunction-associated steatohepatitis detection, fibrosis assessment, treatment-response prediction and precision medicine in MASLD.

## 1 Introduction

### 1.1 MASLD as a central model of metabolic dysfunction

Metabolic dysfunction-associated steatotic liver disease (MASLD) is defined by hepatic steatosis in individuals with at least one cardiometabolic risk factor, after exclusion of other specific causes of steatosis and alcohol-dominant liver disease (1). Currently affecting approximately 38% of adults worldwide, its prevalence is projected to exceed 55% by 2040, driven by parallel epidemics of obesity and type 2 diabetes (2, 3). Beyond hepatic injury, MASLD increases the risk of cardiovascular disease, chronic kidney disease, and extrahepatic malignancies through interconnected mechanisms involving inflammation, lipotoxicity, and gut microbiota dysbiosis, positioning it as a systemic metabolic disorder rather than a liver-specific condition (4, 5).

### 1.2 Evolution of MASLD nomenclature

The nomenclature of this condition has evolved in step with mechanistic understanding. The historical term nonalcoholic fatty liver disease (NAFLD) reflected an exclusionary diagnosis; in 2020, metabolic dysfunction-associated fatty liver disease (MAFLD) was proposed to highlight metabolic dysfunction, and in 2023 a multi-society Delphi consensus formally adopted MASLD and metabolic dysfunction-associated steatohepatitis (MASH) (1, 6). This shift from exclusion-based to affirmation-based criteria is more than semantic: over 99.5% of patients previously classified as NAFLD satisfy MASLD criteria, validating the direct applicability of prior research to the current framework (7).

### 1.3 Limitations of current MASLD biomarkers

Despite this conceptual progress, non-invasive diagnosis remains a critical bottleneck. Liver biopsy remains the histological reference standard, but its invasiveness, sampling variability, and limited scalability preclude population-level application (8, 9). Established non-invasive tools, including fibrosis index-4 (FIB-4), enhanced liver fibrosis test (ELF) and vibration-controlled transient elastography (VCTE), are useful for fibrosis risk stratification and perform best in identifying or excluding advanced fibrosis, but they remain insufficient for reliably detecting active steatohepatitis, hepatocyte ballooning, or earlier fibrotic transition (9, 10). Recent prognostic models further extend the role of non-invasive testing from fibrosis staging to clinical event prediction. For example, the ANTICIPATE-NASH models, which combine liver stiffness measurement, platelet count and body mass index, stratified the risk of liver-related events better than histological fibrosis stage in patients with F3-F4 MASLD and compensated advanced chronic liver disease (11). More fundamentally, these tools capture structural or indirect biochemical changes without resolving the molecular heterogeneity that underlies divergent clinical trajectories among patients with similar histological phenotypes.

### 1.4 Emergence of multi-omics approaches

Multi-omics technologies provide a mechanistically grounded framework for investigating MASLD heterogeneity. Recent reviews have comprehensively summarized omics-based biomarker discovery, the methodological barriers to clinical translation, and the mechanistic insights provided by single-cell and spatial technologies across MASLD and related steatotic liver diseases (12–14). However, an important gap remains between molecular characterization and clinically meaningful stratification. In particular, it remains unclear how different omics approaches relate to distinct dimensions of MASLD heterogeneity, which molecular findings represent exploratory associations or mechanistic signatures rather than clinically validated biomarkers, and whether they provide incremental value beyond established clinical and non-invasive assessment.

The present Review therefore takes a MASLD-specific and clinically anchored approach. We integrate single-omics, single-cell, spatial and integrative multi-omics evidence within a framework centred on hepatic progression, cardiometabolic risk and treatment-response heterogeneity. Candidate molecular signatures are evaluated according to their intended clinical use and level of validation, with particular attention to whether available evidence demonstrates incremental value beyond conventional assessment. This framework links molecular resolution with practical questions in risk stratification, at-risk MASH detection, fibrosis assessment, prognosis and treatment-response evaluation. Here and throughout this Review, “at-risk MASH” generally refers to histologically confirmed MASH with a disease activity score of ≥4 and significant fibrosis (≥F2). However, the exact criteria vary across studies; some additionally require at least one point in each histological activity component or exclude cirrhosis (15, 16).

### 1.5 Literature search strategy

A structured literature search was conducted primarily in PubMed/MEDLINE and Web of Science for studies published between 2010 and 2026. Search terms combined disease-related concepts, including “metabolic dysfunction-associated steatotic liver disease,” “MASLD,” “metabolic dysfunction-associated steatohepatitis,” “MASH,” “nonalcoholic fatty liver disease,” “NAFLD,” “nonalcoholic steatohepatitis,” and “NASH,” with omics-related concepts, including genomics, genome-wide association studies, polygenic risk scores, transcriptomics, single-cell RNA sequencing, spatial transcriptomics, proteomics, lipidomics, metabolomics, multi-omics, and integrative omics. Additional targeted searches were performed for artificial intelligence, machine learning, biomarker validation, treatment response, and precision medicine. Priority was given to human studies, well-characterized cohorts, externally validated biomarker studies, prospective studies, clinical trials, and relevant guidelines. Earlier landmark studies published before 2010 were also included when necessary to provide essential mechanistic or historical context. As this was a narrative Review, study selection was based on relevance to the scope and translational focus of the manuscript rather than on a formal systematic-review protocol.

## 2 Pathophysiology and heterogeneity of MASLD

MASLD encompasses a dynamic continuum from isolated hepatic steatosis to metabolic dysfunction-associated steatohepatitis (MASH), progressive fibrosis, cirrhosis and hepatocellular carcinoma (17, 18). However, progression is neither linear nor inevitable: many patients remain at the steatosis stage, whereas others advance rapidly to fibrotic disease, and HCC may arise even in the absence of established cirrhosis (19, 20). Importantly, MASLD heterogeneity extends beyond hepatic disease progression. Patients with apparently similar liver phenotypes may differ in their future liver-related outcomes, systemic cardiometabolic risk and, potentially, response to treatment. These dimensions are clinically related but not interchangeable and may therefore require different approaches to risk stratification. The key clinical challenge is not merely to detect hepatic fat or assign a histological stage, but to determine which sources of heterogeneity are already captured by conventional assessment and where additional molecular information may be informative (21).

Importantly, clinical heterogeneity does not by itself imply a need for omics-based classification. Established clinical variables and non-invasive tools already provide meaningful stratification for several outcomes. For example, the ANTICIPATE-NASH models predict liver-related events in patients with advanced MASLD using liver stiffness, platelet count and body mass index (11). Similarly, Raverdy et al. identified liver-specific and cardiometabolic MASLD subtypes using six routinely available variables, including age, BMI, HbA1c, ALT, LDL cholesterol and triglycerides (22). Despite comparable baseline liver severity, these subtypes showed divergent clinical trajectories. Importantly, this data-driven classification has subsequently been validated in three large Asian cohorts, in which the cardiometabolic subtype consistently showed greater cardiovascular risk, whereas the liver-specific subtype was more strongly associated with liver-related events (23). Molecular and genetic analyses helped characterize the biological differences underlying these phenotypes but were not required for their initial clinical identification. Complementary genetic evidence from partitioned polygenic risk scores further supports the existence of liver-predominant and systemic cardiometabolic forms of MASLD (24). Recent precision-subtyping frameworks therefore increasingly view clinical phenotyping and molecular profiling as complementary rather than competing approaches (25). Together, these findings indicate that clinically meaningful heterogeneity can sometimes be recognized using accessible clinical information, whereas molecular profiling may provide an additional layer of biological interpretation and, potentially, further risk refinement.

At the mechanistic level, this heterogeneity reflects variation in genetic susceptibility, metabolic phenotype, gut–liver signalling, immune-cell remodelling and fibrogenic responses (26, 27). Omics approaches interrogate these processes at complementary levels: genomics captures inherited susceptibility; transcriptomic, single-cell and spatial approaches resolve active pathways, cellular states and tissue niches; proteomics reflects circulating disease activity and matrix remodelling; lipidomics characterizes disease-relevant lipid composition; and metabolomics captures mitochondrial, bile acid and gut–liver metabolic disturbances. Their strongest current contribution is therefore mechanistic resolution of clinically observed heterogeneity. Demonstrating additional clinical value, however, requires more than identifying molecular differences: candidate signatures must show reproducible improvement in disease classification, risk prediction or treatment-related decision-making beyond established clinical and non-invasive assessment. The following sections therefore first examine evidence from individual omics layers and subsequently consider whether integrative multi-omics approaches provide additional biological or clinically actionable information. This conceptual framework, linking the MASLD disease spectrum with complementary omics layers and their potential clinical applications, is summarized in Figure 1.

**Figure 1:**
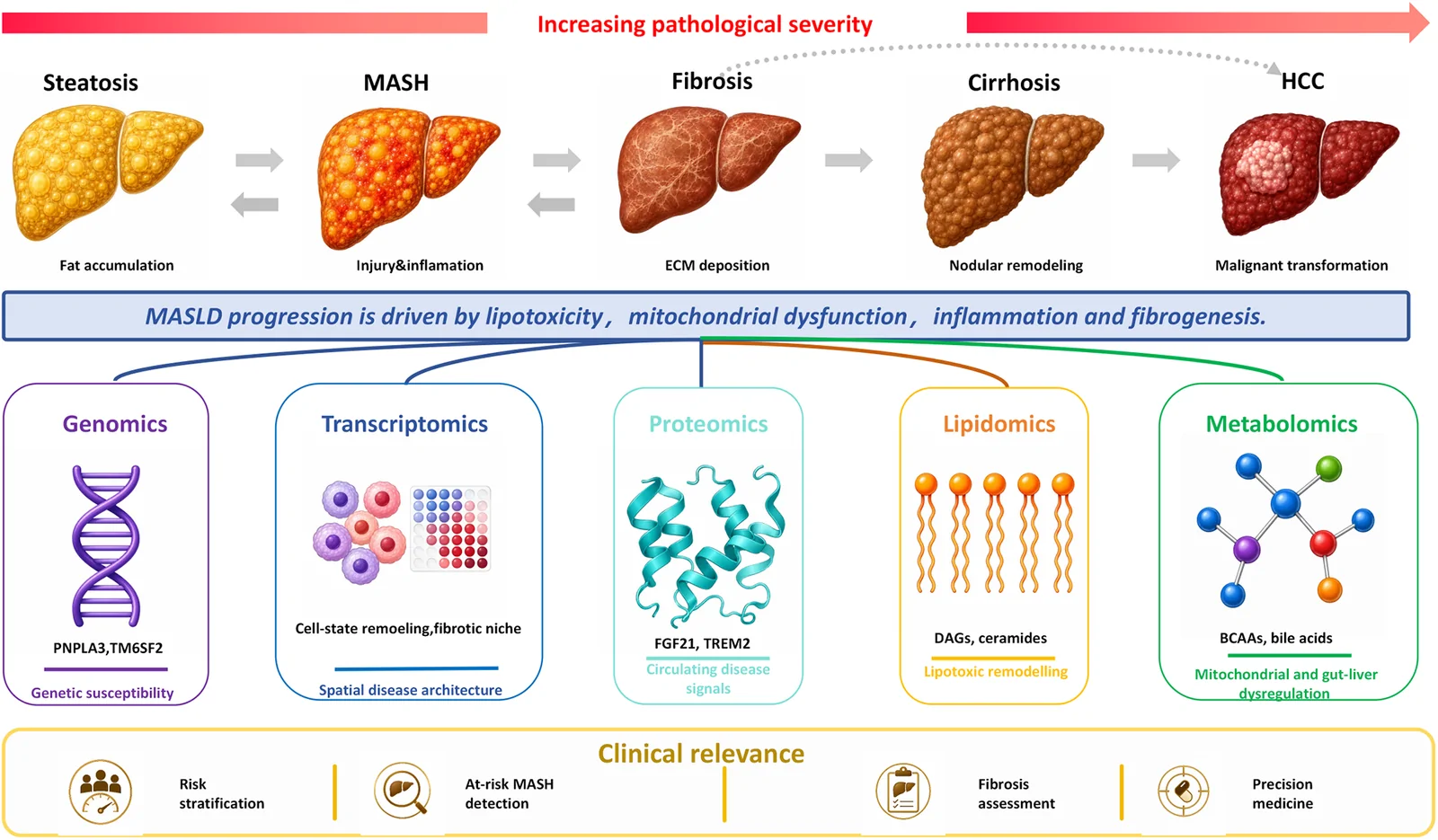
Multi-omics landscape across the heterogeneous natural history of MASLD. MASLD follows heterogeneous and non-linear trajectories. Patients may remain stable, progress or partially regress across steatosis, MASH and fibrosis, while hepatocellular carcinoma may develop through cirrhotic or non-cirrhotic pathways. Genomics, transcriptomics/single-cell/spatial omics, proteomics, lipidomics and metabolomics capture complementary molecular layers of this continuum, supporting risk stratification, at-risk MASH detection, fibrosis assessment and precision medicine. BCAAs, branched-chain amino acids; DAGs, diacylglycerols; ECM, extracellular matrix; FGF21, fibroblast growth factor 21; HCC, hepatocellular carcinoma; MASLD, metabolic dysfunction-associated steatotic liver disease; MASH, metabolic dysfunction-associated steatohepatitis; PNPLA3, patatin-like phospholipase domain-containing protein 3; TM6SF2, transmembrane 6 superfamily member 2; TREM2, triggering receptor expressed on myeloid cells 2.

## 3 Omics-derived molecular signatures in MASLD

### 3.1 Genomic biomarkers

Genomic studies have reshaped our understanding of MASLD. Although MASLD is primarily driven by metabolic stress, its susceptibility, histological severity and clinical trajectory are strongly modified by inherited genetic variation (28, 29). Genome-wide association studies (GWAS) have identified several reproducible loci, including *PNPLA3*, *TM6SF2, GCKR, MBOAT7* and *HSD17B13*. These loci do not act through a single pathway. Instead, they highlight several complementary biological axes: lipid droplet remodelling, hepatic lipid export, glucose-driven *de novo* lipogenesis, phospholipid remodelling and hepatocellular injury responses.

The strongest genetic signal remains *PNPLA3* rs738409 (p.I148M). This variant is closely linked to hepatic triglyceride accumulation and disease severity (28). PNPLA3 participates in lipid droplet metabolism, and the I148M substitution impairs normal lipid mobilization. Recent human-cell-based evidence further extends this mechanism. In primary human hepatocytes and induced pluripotent stem cell (iPSC)-derived hepatocytes, PNPLA3-I148M rewired lipid metabolism, increased endoplasmic reticulum stress, impaired mitochondrial function, enhanced peroxisomal *β*-oxidation and promoted ferroptosis-associated cell death (30). These findings connect a common GWAS locus with hepatocyte injury pathways beyond simple lipid retention. However, the clinical relevance of ferroptosis in PNPLA3-mediated MASLD progression still requires further validation.

Other susceptibility loci define different routes to hepatic fat accumulation*. TM6SF2* rs58542926 (p. E167 K) mainly reflects impaired lipid export. Loss of TM6SF2 function reduces hepatic VLDL secretion and favours intrahepatic triglyceride retention (31, 32). In contrast, *GCKR* variants link MASLD to altered glucose handling and increased substrate availability for *de novo* lipogenesis. In biopsy-proven MASLD, *GCKR* rs780094 was associated with significant fibrosis and higher serum triglyceride levels, suggesting that inherited changes in glucose–lipid flux may influence both steatosis and disease severity (33). *MBOAT7* rs641738 further extends this model to phosphatidylinositol remodelling; and this variant has been associated with MASLD development and severity, while experimental evidence indicates that MBOAT7 loss may promote lysophosphatidylinositol accumulation, inflammatory signalling and fibrogenic remodelling under metabolic stress (34). By contrast, *HSD17B13* provides an example of a protective genetic biomarker where the protein-truncating rs72613567 variant is associated with reduced chronic liver disease risk and lower progression from steatosis to steatohepatitis (35). Mechanistic work further suggests that HSD17B13 inhibition may protect against fibrosis through altered pyrimidine catabolism (36). In biopsy-proven MASLD, combined analysis of *PNPLA3, TM6SF2* and *HSD17B13* variants showed that genetic risk factors were associated with disease severity and could improve prediction of advanced fibrosis when incorporated into risk models (37).

Clinically, the value of genomic biomarkers may lie more in cumulative genetic risk assessment than in isolated single-variant testing. Polygenic risk scores (PRS) integrate the effects of multiple susceptibility variants and may therefore provide a broader estimate of inherited risk. PRS incorporating established MASLD-associated loci have been evaluated for susceptibility, fibrosis progression and longer-term liver-related outcomes. Bianco et al. showed that a PRS based on MASLD-related variants improved non-invasive stratification of hepatocellular carcinoma (HCC) risk in individuals with metabolic dysfunction (38). More recently, partitioned PRS have extended this concept beyond aggregate genetic risk. Jamialahmadi et al. identified genetically distinct liver-predominant and systemic cardiometabolic MASLD patterns, suggesting that polygenic architecture may help distinguish different dimensions of disease risk rather than simply indicating overall susceptibility (24). Importantly, the clinical value of PRS depends on whether genetic information provides meaningful incremental information beyond established clinical models. In a cohort of 637 patients with biopsy-proven MASLD, incorporation of *PNPLA3* rs738409 and *TM6SF2* rs58542926 into existing non-invasive fibrosis models produced modest but measurable improvements, particularly among patients with type 2 diabetes. In this subgroup, the AUROC increased by 0.053 for the NAFLD fibrosis score and by 0.058 for FIB-4, although gains in net clinical benefit were relatively small (39). Similarly, a large UK Biobank analysis showed that adding a hepatic-fat PRS directly to conventional fibrosis scores did not improve overall AUROC for severe liver disease. However, PRS-based population stratification improved the performance of FIB-4, Forns and APRI in genetically high-risk groups, particularly for HCC (40). These findings suggest that PRS may be more useful for targeted risk enrichment and subgroup definition than as a universal additive component of existing clinical models.

Cross-ancestry validity remains an additional barrier to clinical implementation. Although major variants such as *PNPLA3* rs738409 have shown associations with steatotic liver disease across multiple populations, allele frequencies and effect estimates vary substantially by ancestry. For example, within a large US Hispanic/Latino cohort, the frequency of the *PNPLA3* rs738409 risk allele ranged from 52% in individuals of Mexican background to 23% in those of Dominican background (41). At the polygenic level, differences in allele frequency, linkage disequilibrium structure, effect-size estimates and the ancestral composition of GWAS training datasets can substantially reduce PRS transferability across populations. Population-specific calibration is therefore required before a PRS developed predominantly in one ancestry can be assumed to provide equivalent risk estimates in another (42).

Overall, genomic biomarkers currently have a complementary rather than replacement role in MASLD assessment. Their strongest near-term value lies in risk enrichment, biologically informed patient stratification and research-oriented precision medicine. Routine clinical implementation will require prospective validation, demonstration of clinically meaningful incremental value beyond established non-invasive tools, and robust cross-ancestry calibration.

### 3.2 Transcriptomics and spatial omics

Transcriptomic profiling captures active disease programmes during MASLD progression, including metabolic stress, inflammatory activation and fibrogenic remodelling. As illustrated in Figure 2, transcriptomic evidence can be organized into a stepwise framework: bulk transcriptomics identifies tissue-level pathways, single-cell RNA sequencing assigns these programmes to specific cell states, and spatial transcriptomics localizes them within histological and spatial niches.

**Figure 2:**
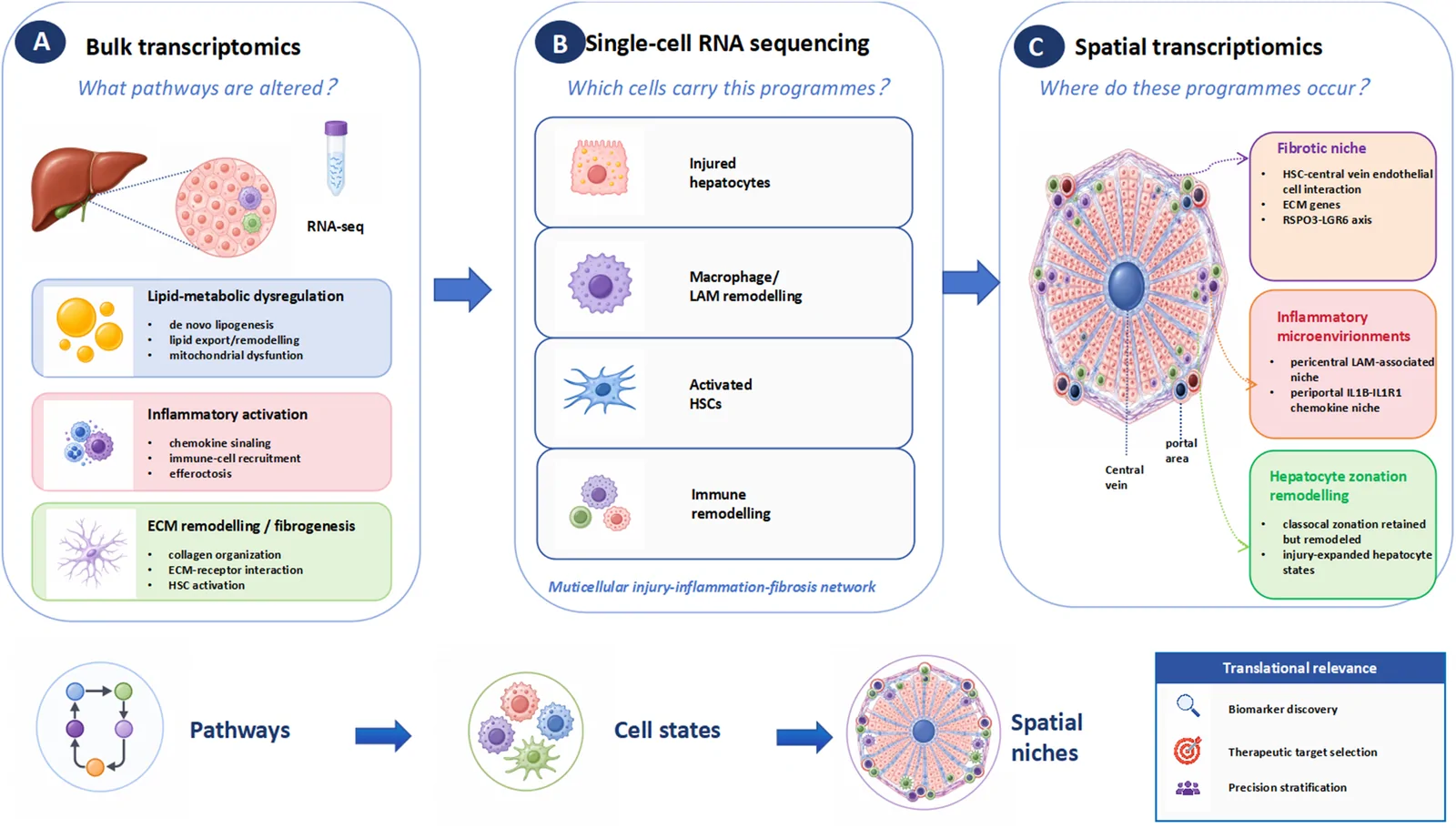
Transcriptomic architecture of MASLD progression. Bulk transcriptomics defines altered tissue-level pathways, single-cell RNA sequencing assigns these programmes to disease-relevant cell states, and spatial transcriptomics localizes them to fibrotic niches, inflammatory microenvironments and remodeled hepatocyte zones. Together, these approaches link pathway dysregulation to cellular remodelling and spatially organized liver injury in MASLD. HSCs, hepatic stellate cells; IL1B, interleukin-1 beta; IL1R1, interleukin-1 receptor type 1; LAMs, lipid-associated macrophages; LGR6, leucine-rich repeat-containing G protein-coupled receptor 6; RNA-Seq, RNA sequencing; RSPO3, R-spondin 3; scRNA-Seq, single-cell RNA sequencing.

#### 3.2.1 Bulk transcriptomics

Bulk transcriptomics links histological progression in MASLD to disease-relevant hepatic transcriptional programmes. Its main mechanistic value lies in defining three interconnected axes: lipid-metabolic dysregulation, inflammatory activation and ECM remodelling.

Dysregulated lipid metabolism represents an early transcriptional feature of MASLD progression. Human liver transcriptomic studies have identified dysregulated expression of lipogenic regulators, lipid droplet and export genes, and cholesterol biosynthetic enzymes, including SREBF1, PNPLA3, TM6SF2 and FDFT1, which are associated with worsening steatosis and liver injury (43). Large-scale RNA-seq further showed that metabolic transcriptional alterations are embedded across the MASLD disease spectrum (44). At the pathway level, transcriptomics-driven metabolic modelling further reveals coordinated alterations in triglyceride biosynthesis, glycerolipid and glycerophospholipid metabolism, fatty acid activation, *β*-oxidation, cholesterol metabolism, bile acid recycling and oxidative phosphorylation (45). These findings suggest that MASLD/MASH is not simply a disease of lipid accumulation, but a disorder of altered lipid handling and impaired metabolic adaptation.

Inflammatory activation forms the second major transcriptomic layer. Interspecies transcriptomic profiling suggests that MASLD progression follows a stepwise transition from metabolic disturbance to inflammatory activation and subsequently to ECM remodelling (46). Consistently, large-scale RNA sequencing across the MASLD spectrum shows that inflammation-related transcriptional changes emerge early after hepatic steatosis onset and become increasingly aligned with hepatocyte ballooning and fibrosis during fibrosing steatohepatitis (44). High-risk transcriptomic profiles are enriched for chemokine signalling, cytokine-cytokine receptor interaction, leukocyte migration, Fc*γ* receptor-mediated phagocytosis, efferocytosis and adaptive immune signalling (47). Recurrent mediators, including CCL19, CCL21, CXCL9, CXCL10, CCL20, IL32, SPP1, TNFRSF12A and GDF15, further connect immune-cell recruitment with lobular inflammation, hepatocyte injury and fibrotic progression (43, 44, 46, 48). Additional evidence from adults with obesity supports this disease-stage pattern. In this high-risk population, MASH without fibrosis was characterized by inflammatory gene expression, whereas fibrotic MASH showed stronger scar- and tissue-remodelling programmes (49).

ECM remodelling represents the transcriptional transition from chronic injury to progressive fibrosis. Fibrosis-associated transcriptional programmes involve collagen organization, focal adhesion, ECM-receptor interaction, PI3K-Akt signalling, Wnt signalling and hepatic stellate cell (HSC) activation. With fibrosis progression, matrix-related genes such as COL1A1, COL1A2, COL14A1, DPT, LUM and TGFB1 become increasingly upregulated, reflecting expansion of a fibrogenic mesenchymal programme (44). Hepatic and visceral adipose tissue transcriptomic data further show that severe MASLD is associated with inflammation, ECM remodelling, mitochondrial dysfunction, reduced autophagy and impaired oxidative phosphorylation (49). Importantly, paired liver transcriptomics and circulating proteomics have translated matrix remodelling into blood-accessible signatures, including ADAMTSL2, THBS2, MFAP4, IGFBP7 and TREM2, which are associated with advanced fibrosis or at-risk MASH (50).

Overall, bulk transcriptomics defines disease-relevant pathways, but it cannot determine which cell types generate these signals or how they interact during progression.

#### 3.2.2 Single-cell RNA sequencing

Single-cell RNA sequencing extends MASLD transcriptomics from tissue-level pathway discovery to cell-specific interpretation (51). In human MASH, single-cell profiling has revealed macrophage activation, distinct activated HSC states and hepatocyte-centred programmes linked to inflammation, fibrosis and complement dysregulation (52). These findings suggest that MASLD progression is not driven by one pathogenic cell type alone. Instead, it reflects coordinated multicellular remodelling involving hepatocytes, macrophages, HSCs, endothelial cells and infiltrating immune cells. Hepatocytes are central initiators of MASLD-related cellular remodelling. Under lipid overload, hepatocytes develop metabolic stress, mitochondrial dysfunction and injury-associated transcriptional programmes. Single-cell and single-nucleus approaches show that lipid-laden or metabolically stressed hepatocytes display reduced fatty acid metabolism, immune activation and tissue-remodelling signals. These findings indicate that hepatocytes are not passive targets of injury. They actively connect metabolic overload to downstream inflammatory and fibrogenic responses.

Macrophage remodelling provides a key bridge between hepatocyte injury and fibrosis. Lipid overload and hepatocyte-derived stress signals activate resident Kupffer cells and promote recruitment of monocyte-derived macrophages (53). Single-cell analyses show that this process is accompanied by rewiring of intrahepatic cellular crosstalk and the emergence of TREM2-positive or lipid-associated macrophage (LAM)-like populations (54, 55). Human MASH scRNA-seq identified inflammatory macrophage subsets enriched for S100A8, S100A9, S100A12 and VCAN, whereas later liver-blood immune atlases described TREM2-positive, S100A9-positive and LAM-like populations associated with liver dysfunction and fibrogenesis (52, 55). Dynamic changes between resident and recruited macrophage populations further support the view that these macrophages represent disease-induced functional states shaped by lipotoxicity, inflammation and fibrosis stage (56). HSC activation represents the fibrogenic arm of this multicellular response. scRNA-seq distinguishes quiescent HSCs from ACTA2-positive myofibroblast-like states and RBP1-positive collagen-producing HSC subsets (52). These activated HSC states interact with macrophages and endothelial cells through profibrogenic pathways, supporting the concept of a multicellular fibrotic niche involving scar-associated macrophages, endothelial cells and collagen-producing mesenchymal cells (57). Immune-cell atlases further reveal expanded hepatic regulatory T cells, monocytic myeloid-derived suppressor cells, dendritic-cell subsets, exhausted CD8-positive T cells and enhanced NK-cell cytotoxicity during MASLD/MASH progression (55, 58).

Overall, scRNA-seq reframes MASLD as an injury-inflammation-fibrosis network. However, because it loses tissue architecture, it cannot determine where these cell states and interactions occur within the liver.

#### 3.2.3 Spatial transcriptomics

Spatial transcriptomics addresses this limitation by preserving anatomical context (59). This is particularly important in MASLD because liver injury is spatially organized rather than uniformly distributed. By adding a “where” dimension to bulk and single-cell transcriptomics, spatial approaches link molecular programmes to fibrotic niches, inflammatory microenvironments and hepatocyte zonation remodelling. Fibrotic niche mapping is one of the clearest applications. In a 61-sample human MASLD spatial multi-omics atlas, Li et al. identified a fibrosis-associated spatial programme enriched in advanced MASH (60). This programme was marked by extracellular-matrix and fibrogenic genes, including COL1A1, COL1A2, THY1, LUM, LOXL1 and MFAP4, and spatially overlapped with inferred HSC and central-vein endothelial-cell abundance. Ligand-receptor analysis further suggested enrichment of the RSPO3-LGR6 axis within fibrotic regions, indicating a potential endothelial cell-HSC interaction that may support focal fibrogenesis (60). Visium profiling of MASLD liver biopsies similarly showed enrichment of ECM-receptor interaction and inflammatory pathways in fibrotic regions, with late fibrosis associated with immunoglobulin induction, senescence-related signals and glycolytic reprogramming (61). These findings indicate that spatial omics can define where fibrosis develops, which cells sustain it and which local signalling axes may drive progression. Spatial transcriptomics also reveals regional inflammatory microenvironments. In the human MASLD atlas, LAMs increased with disease severity and were preferentially enriched in pericentral regions, where they displayed lipid-handling and immunometabolic programmes involving PPAR signalling, fatty acid oxidation and TREM2-associated features (60). In contrast, Fukumoto et al. described a periportal inflammatory niche in which macrophage-derived IL-1β activates IL1R1 on liver sinusoidal endothelial cells, inducing CCL2 and CXCL10 (62). Experimental disruption of this axis reduced inflammatory infiltration and fibrosis in mice (62). Together, these studies suggest that MASLD inflammation is spatially heterogeneous, involving both pericentral LAM-associated immunometabolic niches and periportal IL1B-IL1R1-driven chemokine niches. A third application is hepatocyte zonation remodelling. In a mouse NASH model, Zhou et al. showed that Wnt-target genes were spatially altered and that the Wnt activator Rspo3 expanded from the perivenous region toward the periportal compartment. However, experimental modulation of Rspo3 changed Wnt-target gene distribution without significantly affecting steatosis, inflammation or fibrosis, suggesting that zonation disturbance may be a spatial readout rather than a dominant disease driver (63). Human studies further indicate that MASLD-related hepatocyte changes are superimposed on, rather than simply replacing, classical lobular zonation. Watson et al. confirmed canonical zone 1–3 hepatocyte gradients but identified injury-expanded hepatocyte states in fibrotic liver that were not restricted to a specific zonal compartment (64). Yakubovsky et al. showed that early steatotic hepatocytes already exhibit mitochondrial remodelling before overt MASH or fibrosis, indicating that disease-associated hepatocyte reprogramming may begin early (65). Together, these findings suggest that MASLD reshapes classical metabolic zonation while generating additional disease-associated hepatocyte states. Overall, spatial transcriptomics connects molecular signals, cell states and tissue architecture. It shows that MASLD progression is organized within fibrotic niches, inflammatory microenvironments and remodelled hepatocyte zones. Although its current value remains mainly mechanistic, spatially restricted disease programmes may help refine future biomarker discovery, therapeutic target selection and precision stratification in MASLD.

### 3.3 Proteomic biomarkers

Proteomics provides a translational bridge between liver biology and blood-accessible biomarkers in MASLD. Compared with genomics, circulating proteins are more dynamic. Compared with liver transcriptomics, they are easier to adapt to non-invasive testing. Therefore, proteomics is well suited for detecting active steatohepatitis, fibrotic progression and systemic metabolic risk.

Several circulating proteins reflect key biological processes in MASLD. Fibroblast growth factor 21 (FGF21) is a liver-enriched stress-responsive hepatokine. It reflects hepatic metabolic stress and disease activity. In MASLD, hepatic FGF21 expression and circulating FGF21 levels tend to increase from simple steatosis to MASH (66–68). However, its association with fibrosis is less consistent. FGF21 should therefore be viewed as an activity- or stress-related signal, rather than a dedicated fibrosis biomarker.

Adiponectin provides an adipose-derived protective signal. Lower adiponectin levels may reflect adipose dysfunction, insulin resistance and loss of anti-inflammatory protection. They have also been associated with more advanced fibrosis, especially when combined with clinical variables (69, 70). Fetuin-A represents another metabolic-inflammator*y* axis. As a liver-derived glycoprotein, fetuin-A can facilitate free-fatty-acid-induced TLR4 activation, thereby promoting inflammatory signalling and insulin resistance (71, 72).

Two additional hepatokines link lipid metabolism and inflammatory amplification in MASLD. Angiopoietin-like protein 8 (ANGPTL8) inhibits the PGC-1*α*/PPAR*α* axis, worsening hepatic lipid accumulation, and activates NF-*κ*B-driven inflammatory signalling, creating a feed-forward loop that accelerates disease progression (71, 73). Retinol-binding protein 4 (RBP4), particularly in its exosomal form derived from hepatocytes, promotes inflammatory polarization of Kupffer cells through the NOX2/ROS/NF-*κ*B pathway, which in turn stimulates lipogenesis and impairs fatty acid oxidation via JAK2/STAT3 signalling (74). Nevertheless, these candidate proteins are not MASLD-specific and are strongly influenced by systemic metabolic context, including obesity, type 2 diabetes, adipose tissue dysfunction and low-grade inflammation.

Several representative panels illustrate this translational potential. Serum proteomic signatures can capture histological features such as steatosis, inflammation, ballooning and fibrosis (75). Proteo-transcriptomic approaches further link circulating proteins with intrahepatic disease biology and can identify active steatohepatitis and advanced fibrosis (50). More clinically oriented panels, such as N3-MASH, TLM3 and APASHA, aim to support MASH detection, fibrosis stratification or risk enrichment in metabolically high-risk populations (76–78). Additional protein-based models extend proteomics toward incident SLD/MASLD prediction and systemic complication risk (66, 79).

Representative protein-based and proteomics-derived panels are summarized in Table 1. Overall, these panels show that proteomics is not only a discovery platform. It can also support clinically relevant risk stratification for steatohepatitis activity, fibrosis progression and metabolic complications. Nevertheless, most panels still require broader external validation, assay standardization, cost-effectiveness assessment and clearly defined clinical thresholds before routine implementation.

**Table 1:** Representative protein-based and proteomics-derived panels for MASLD/MASH clinical stratification.

Panel/signature	Model composition	Main clinical role	Translational positioning
Sanyal serum proteomic signature	Modified-aptamer serum proteomics; endpoint-specific protein models for steatosis, lobular inflammation, ballooning and fibrosis	Assessment of histological activity and fibrosis	Foundational evidence that serum proteomics can reflect liver histology (75)
Govaere proteo-transcriptomic signature	4,730 circulating proteins plus paired liver transcriptomics; 31-marker signature; compact model including ADAMTSL2, AKR1B10, CFHR4, TREM2, BMI and T2DM status	Active steatohepatitis and advanced fibrosis stratification	Links circulating proteins with intrahepatic disease biology (50)
ADAMTSL2/NFPP	ADAMTSL2 alone; NFPP composed of ACY1, ADAMTSL2, ADH4, ALDOC, ASL, ENPP7, FBP1 and FTCD	Detection of at-risk NASH and significant fibrosis	Protein fibrosis panel with performance comparable or better to standard fibrosis scores in validation cohorts (115)
N3-MASH	CXCL10, CK-18 M30 and adjusted BMI	Non-invasive diagnosis of MASH	Compact protein-based blood panel for active MASH detection and biopsy enrichment (76)
TLM3	IGFBP7, SSC5D and SEMA4D	Detection and staging of MASLD-related fibrosis	Mechanism-informed fibrosis panel reflecting active fibrogenesis (77)
APASHA	APOF, PCSK9, AFM, S100A6, HbA1c and AZGP1	MASH risk stratification in individuals with obesity	Integrates liver-secreted and plasma protein signals (78)
MSI-F	GDF15, decorin and clinical/metabolic variables	Prediction of MASLD-related hepatic fibrosis	Reflects metabolic stress and ECM remodelling (137)
RBP4-FGF21-adiponectin-osteocalcin model	RBP4, FGF21, adiponectin and osteocalcin	Incident SLD/MASLD and metabolic risk prediction	Links circulating metabolic proteins with systemic metabolic risk (66)
MASH Dx	THBS2, GDF15, SELE and IGFBP7 plus age, BMI, ALT, diabetes and hypertension	Identification of MASH and high-risk MASH with F2 + fibrosis	Serum protein risk stratification score for referral enrichment and diagnostic triage (156)
Organ damage proteomic signature/ADGRG1-related signature	Prioritized organ-damage proteomic panel; ADGRG1/GPR56 highlighted as a key deregulated protein	Progressive disease, significant fibrosis and systemic complication risk	Extends proteomics from liver staging to systemic risk stratification (79)

ADGRG1, adhesion G protein-coupled receptor G1; AFM, afamin; ALT, alanine aminotransferase; ANGPTL8, angiopoietin-like protein 8; APOF, apolipoprotein F; AZGP1, zinc-alpha-2-glycoprotein; BMI, body mass index; CK-18 M30, caspase-cleaved cytokeratin-18 fragment M30; FGF21, fibroblast growth factor 21; MASLD, metabolic dysfunction-associated steatotic liver disease; MASH, metabolic dysfunction-associated steatohepatitis; MSI-F, metabolic stress index-fibrosis; NFPP, NAFLD fibrosis protein panel; RBP4, retinol-binding protein 4; SLD, steatotic liver disease; T2DM, type 2 diabetes mellitus.

### 3.4 Lipidomics in MASLD

Lipidomics refines the understanding of MASLD by focusing not only on hepatic fat burden but also on disease-relevant lipid composition. Excess fatty acid influx, *de novo* lipogenesis, impaired oxidation and altered VLDL export reshape hepatic and circulating lipid pools, producing lipotoxic species that promote insulin resistance, mitochondrial stress, inflammation and fibrogenesis (80, 81).

Sphingolipids, including ceramides and sphingomyelins, represent an important bioactive lipid axis in MASLD. Ceramide-related pathways have been implicated in insulin-signalling disruption, mitochondrial dysfunction, endoplasmic reticulum stress, inflammation and apoptosis (80, 81). However, their clinical relevance appears to depend on specific lipid species rather than total sphingolipid abundance. In a liver biopsy-based lipidomics study of 365 individuals, Vvedenskaya et al. quantified 316 lipid species across 22 lipid classes and found progressive accumulation of diacylglycerols (DAGs), triacylglycerols and cholesteryl esters in MASLD/MASH, whereas the bulk abundance of glycerophospholipids and sphingolipids remained relatively stable. Notably, selected sphingomyelin species, including SM 43:3;2 and SM 43:1;2, helped distinguish steatotic and non-steatotic liver lipidomes, suggesting species-level value for disease stratification (82).

DAGs provide another mechanistic link between lipid overload and metabolic dysfunction. Beyond serving as intermediates in triglyceride synthesis, DAGs act as signalling lipids and may impair hepatic insulin signalling through PKC*ε*-related pathways (81). Velenosi et al. showed that patients with MASLD exhibited a sustained postprandial increase in multiple plasma DAG species, mainly within liver-derived VLDL particles rather than meal-derived lipids. This response correlated with insulin levels and appeared early in disease development, suggesting that circulating DAGs may reflect altered hepatic lipid handling and insulin resistance rather than directly indicating fibrosis severity (83).

Phosphatidylcholines (PCs) and related glycerophospholipids provide complementary information on membrane remodelling, lipoprotein assembly and hepatocellular injury. Plasma lipidomic studies have shown that individuals with MASLD exhibit higher concentrations of sphingolipids, glycerolipids and glycerophospholipids than MASLD-free subjects. Among MASLD patients, selected lipid species, particularly PC(36:2), may help distinguish MASH from non-MASH MASLD (84). Similarly, a serum sterol and lipidomic signature combining lipid species with ALT showed strong performance for identifying MASH, supporting the potential of PC- and sterol-related signatures in detecting steatohepatitis (85).

Clinically, the main value of lipidomics lies in multi-lipid panels rather than isolated biomarkers. Lipidomic panels integrate storage lipids, membrane lipids, sterol metabolism and sphingolipid signalling, thereby improving the prediction of MASH and fibrosis risk. A lipidomics-driven machine-learning model incorporating clinical variables and lipid species such as Lysophosphatidylcholine 20:4 (LPC 20:4), Monoalkylglycerophosphocholine O-16:1(LPC O-16:1), Lysophosphatidylethanolamine 18:0 (LPE 18:0), Diacylglycerol 18:1–18:2 (DG 18:1–18:2) and Cholesterol Ester 20:4 (CE 20:4) achieved good discrimination for MASH (86). More clinically, the metabolomics-advanced steatohepatitis fibrosis score (MASEF), based on 12 serum lipids plus BMI, AST and ALT, was developed in 790 patients and validated in 565 patients to identify at-risk MASH, achieving AUCs of 0.76 and 0.79 in the derivation and validation cohorts, respectively (16).

Overall, lipidomics reframes MASLD as a disorder of lipid quality rather than lipid quantity alone. Sphingolipid-related species may reflect lipotoxic stress, DAGs capture altered hepatic lipid export and insulin resistance, and PC- or sterol-related signatures may help identify steatohepatitis. Its greatest clinical potential lies in validated multi-lipid panels for MASH and fibrosis-risk stratification.

### 3.5 Metabolomics signatures

Across human studies, several recurring metabolic signatures have been identified in MASLD, including perturbations in branched-chain amino acids, long-chain acylcarnitines, tricarboxylic acid cycle intermediates, bile acids and gut microbiota-derived metabolites. Branched-chain amino acids (BCAA), including leucine, isoleucine and valine, primarily reflect impaired amino-acid catabolism and systemic insulin resistance in MASLD. Reduced BCAA catabolic activity and altered BCKDK-BCKDHA regulation can promote BCAA accumulation, while excess BCAAs may impair mitochondrial biogenesis, fatty-acid oxidation and insulin signalling through PGC-1*α*, mTORC1/NF-*κ*B and IRS-related pathways (87–89). However, BCAAs are strongly influenced by obesity, diet, diabetes and skeletal-muscle metabolism; therefore, they should be interpreted as systemic insulin-resistance-associated metabolites rather than liver-specific MASLD biomarkers.

Acylcarnitines provide a more direct signal of mitochondrial fatty-acid handling. Under lipid overload, incomplete *β*-oxidation alters short-, medium- and long-chain acylcarnitine profiles, and several plasma acylcarnitines have shown discriminatory value for MASLD or associations with fibrosis progression (90, 91). Nevertheless, acylcarnitine profiles are sensitive to nutritional state and disease stage, and individual species may change in different directions. They are therefore best interpreted as indicators of altered mitochondrial fatty-acid handling rather than uniform single-metabolite markers of fibrosis.

Glycolytic and TCA-cycle-related metabolites link mitochondrial dysfunction to immunometabolic inflammation. Lactate and succinate can accumulate when oxidative metabolism is impaired and may actively shape inflammatory and fibrogenic responses. Lactate has been linked to macrophage activation and HSC metabolism, whereas succinate can signal through SUCNR1/GPR91 and HIF-1*α*-related pathways to promote inflammatory cytokine production and tissue remodelling (92–97). These metabolites therefore provide mechanistic insight into how mitochondrial stress may amplify inflammation and fibrosis, although their circulating levels are not specific to MASLD.

Bile acids represent a gut–liver axis-related metabolic signature in MASLD (98). In MASLD/MASH,

circulating total bile acids are often increased, accompanied by shifts in conjugated bile acid species and changes in the composition of the bile acid pool (99, 100). Mechanistically, these alterations may reflect impaired FXR–FGF19 feedback, leading to insufficient suppression of CYP7A1-mediated bile acid synthesis, together with altered hepatocellular bile acid transport, including increased uptake and reduced canalicular efflux (101–103). This imbalance may promote intrahepatic bile acid accumulation, microcholestatic stress, mitochondrial dysfunction and hepatocyte injury. However, bile acid profiles are strongly shaped by insulin resistance, gut microbiota, diet and disease severity. Therefore, bile acids should be viewed as context-dependent biomarkers of gut-liver metabolic dysregulation rather than liver-specific markers of MASH alone (104).

Microbial metabolites provide an additional gut-liver axis layer in MASLD metabolomics. Beyond bile acids, microbiota-related co-metabolites include short-chain fatty acids, hippurate, indole derivatives and other amino-acid-derived metabolites (105). These metabolites may influence intestinal barrier integrity, endotoxin exposure, immune activation and hepatic energy metabolism. Tryptophan-derived indole metabolites are particularly relevant. Gut microbiota-derived indole-3-propionic acid and indole-3-acetic acid are decreased in patients with hepatic steatosis. Experimental supplementation attenuates steatosis and inflammation in MASLD models, partly through reduced endotoxin-related inflammatory signalling (106). These findings suggest that microbial metabolites may link diet, gut microbiota and hepatic inflammation. However, their levels are highly dependent on diet, microbiome composition, intestinal transit and host metabolic status. They should therefore be viewed as context-dependent gut-liver axis biomarkers rather than liver-specific diagnostic markers.

Overall, metabolomics captures disease-proximal metabolic stress in MASLD, particularly impaired amino-acid catabolism, mitochondrial fatty-acid oxidation, immunometabolic signalling, bile acid dysregulation and microbiota-derived gut-liver axis metabolites.

## 4 Multi-omics integration and systems biology

In this Review, single-omics studies are defined as those interrogating one molecular layer, even when the resulting data are analysed using machine learning, pathway analysis or other computational approaches. By contrast, integrative multi-omics refers to studies that jointly analyse two or more distinct omics layers using an explicit integrative analytical framework to identify cross-layer relationships, molecular modules, disease subtypes or composite signatures. Studies that measure multiple omics layers in parallel without explicit cross-layer integration are considered multi-platform profiling rather than true integrative multi-omics. Building on individual omics layers, integrative multi-omics aims to connect genetic, transcriptional, proteomic, lipidomic and metabolic signals into coherent disease modules. This is particularly important in MASLD because histologically similar patients may differ substantially in genetic susceptibility, inflammatory activation, fibrogenic remodelling and systemic metabolic risk. Therefore, the main purpose of integration is not simply to combine more biomarkers, but to identify biologically meaningful disease states that cannot be resolved by a single molecular layer. As illustrated in Figure 3, multi-omics integration in MASLD can be conceptualized as a stepwise framework that links heterogeneous molecular inputs to integrative analysis, computational interpretation, disease-signature generation and clinical translation.

**Figure 3:**
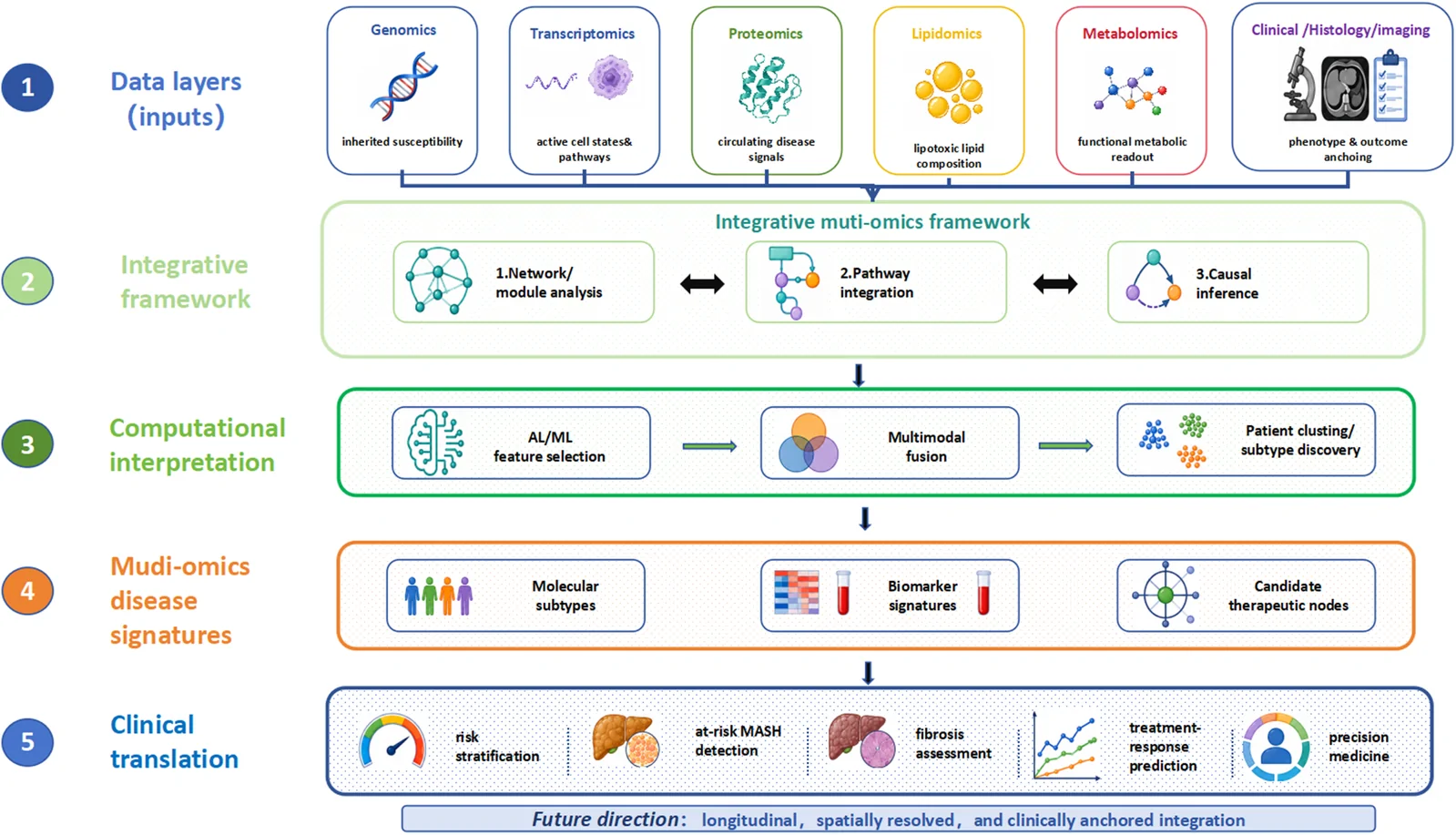
Integrative multi-omics framework for disease-state modelling and clinical translation in MASLD. Multi-omics integration links genomics, transcriptomics, proteomics, lipidomics, metabolomics and clinically anchored phenotypic data into a stepwise translational framework. Network/module analysis, pathway integration and causal inference organize heterogeneous molecular signals into interpretable biological modules. These integrated data are further processed through computational approaches, including AI/ML-based feature selection, multimodal fusion and patient clustering, to derive molecular subtypes, biomarker signatures and candidate therapeutic nodes. Ultimately, these outputs support risk stratification, at-risk MASH detection, fibrosis assessment, treatment-response prediction and precision medicine. The framework also highlights future directions toward longitudinal, spatially resolved and clinically anchored integration. AI, artificial intelligence; ML, machine learning.

### 4.1 Integrative multi-omics frameworks

Network-based and module-based approaches provide one strategy for organizing dispersed molecular alterations. In a Chinese MASLD cohort, Ding et al. integrated WGS, proteomics, phosphoproteomics, lipidomics and metabolomics and classified patients into three molecular subtypes with distinct metabolic, inflammatory-fibrotic and oncogenic programmes (107). This study illustrates how multi-omics can move MASLD classification beyond histological stage toward molecularly defined disease architecture. In parallel, genome-scale metabolic modelling has mapped MASLD transcriptomic alterations onto hepatocyte-specific metabolic networks, identifying serine deficiency and prioritizing PSPH, SHMT1 and BCAT1 as candidate therapeutic nodes (108). These examples show that integration can convert high-dimensional molecular data into interpretable biological modules and candidate intervention points.

Pathway-level integration further links molecular signatures to disease mechanisms. Integrated metabolomic, lipidomic and proteomic profiling of MASH has revealed coordinated alterations in fatty acid uptake, lipid droplet formation, glycolysis, amino acid metabolism and bile acid accumulation (109). Similarly, proteo-transcriptomic analyses have connected circulating proteins with intrahepatic pathological processes, showing that fibrosis-associated proteins are enriched in cell adhesion and inflammatory pathways, whereas activity-associated proteins relate to lipid metabolism, amino acid biosynthesis and bile acid catabolism (50). Causal inference provides an additional layer of interpretation. For example, Mendelian randomization using MASLD GWAS-derived instruments and blood metabolomic data suggested plasma tyrosine as a disease-influenced metabolic biomarker rather than a simple cross-sectional correlate (110–112).

Together, these studies indicate that integrative multi-omics can define molecular modules, prioritize candidate biomarkers and generate mechanistic hypotheses (113). However, most current studies remain cross-sectional and cohort-specific. The frontier of MASLD multi-omics is therefore moving toward longitudinal, spatially resolved and clinically anchored integration, where molecular modules are tested for their ability to predict progression, regression and treatment response.

### 4.2 Computational modelling and AI-based interpretation

Computational modelling and artificial intelligence (AI) provide tools for extracting stable patterns from high-dimensional omics data. Their value in MASLD lies not only in improving predictive accuracy, but also in identifying reproducible molecular features that correspond to biologically meaningful disease states. In this context, AI/ML should be viewed as an interpretative layer between multi-omics discovery and clinical translation.

In biomarker discovery, machine-learning (ML) approaches have been used to integrate clinical variables with molecular profiles. In the IMI DIRECT cohorts, Atabaki-Pasdar et al. combined clinical and multi-omics data using LASSO-based feature selection and random forest modelling (114). Although the full model only modestly improved discrimination over clinically accessible variables alone, the study showed how ML can prioritize molecular features associated with fatty liver disease (114). Similarly, Sanyal et al. used modified-aptamer proteomics from biopsy-confirmed MASLD cohorts to derive separate models for steatosis, lobular inflammation, hepatocellular ballooning and fibrosis, with combined outputs used to identify at-risk MASH and monitor longitudinal change during treatment (75). Corey et al. further identified ADAMTSL2 and an eight-protein soluble signature associated with at-risk NASH and significant fibrosis, demonstrating that ML can compress high-dimensional protein data into more interpretable clinical signatures (115).

AI/ML approaches are also useful for patient stratification. Data-driven clustering has identified clinically relevant MASLD subtypes with similar hepatic phenotypes but different biological profiles and cardiometabolic outcomes (22). Such approaches support a shift from a single histological label toward biologically informed subgroups. However, clustering-derived subtypes require careful external validation because they may be sensitive to cohort composition, variable selection and preprocessing methods.

A more advanced direction is multimodal integration. Conway et al. combined deep learning-based quantitative histopathology with liver transcriptomics and identified a histopathology-driven five-gene expression signature associated with fibrogenesis, disease progression and liver-related events in advanced NASH (116). The enrichment of Notch signalling within this signature illustrates how AI can connect digital pathology with molecular mechanism rather than functioning as a purely predictive black box. Future AI models should move beyond tabular omics prediction toward multimodal integration of digital pathology, imaging-derived phenotypes, clinical trajectories and molecular data. Their clinical value will depend on external validation, calibration, transparent feature selection and evidence that model-guided decisions improve referral, risk stratification or treatment-response prediction beyond established tools (12, 117).

### 4.3 Multi-omics disease signatures

Multi-omics disease signatures represent an intermediate layer between mechanistic discovery and clinical biomarker implementation. They should not be interpreted as ready-to-use clinical tests unless validated in independent cohorts and tested against existing non-invasive tools. Their main value at this stage is to define biologically coherent disease states, particularly steatosis, at-risk MASH, significant fibrosis and progression-prone disease. Early integrated models mainly focused on hepatic steatosis. Multi-domain modelling that combined clinical phenotypes, PNPLA3 genotype and serum proteomics improved non-invasive detection of hepatic steatosis, supporting the principle that molecular and clinical layers provide complementary information (118).

More clinically relevant signatures have shifted toward at-risk MASH and fibrosis. A proteo-transcriptomic strategy integrating 4,730 circulating proteins with paired liver RNA-seq generated a 31-marker signature and a compact model including ADAMTSL2, AKR1B10, CFHR4, TREM2, BMI and T2DM status. The model achieved an AUC of 0.878 in the discovery cohort and 0.80 in an independent validation cohort for detecting at-risk NASH, and significantly outperformed FIB-4, NFS and the AST/ALT ratio. Comparison with FAST was available only in smaller subsets of the cohorts and should therefore be interpreted more cautiously (50). Serum metabolomic panels developed across three independent cohorts also identified significant fibrosis and inflammatory activity, with the fibrosis panel outperforming several established fibrosis scores (119). Cell-resolved and spatially informed signatures further extend this concept from blood-based detection to mechanistic disease stratification. Integrated single-cell and spatial transcriptomic analyses identified DTNA-positive macrophages and the RUNX2-PLG-PARD3 axis as a MASH-associated fibrotic signature, while ensemble machine-learning models distinguished MASLD from MASH with a mean AUC of 0.839 (120). These examples suggest that future disease signatures may combine circulating accessibility with cell-state and spatial specificity. Overall, multi-omics disease signatures should be viewed as a translational bridge rather than a final clinical endpoint. They help organize molecular heterogeneity into biologically interpretable disease states, whereas routine clinical deployment requires standardized assays, external validation, prospective utility testing and comparison with current serum-, imaging- and elastography-based pathways. These implementation issues are discussed further in Section 6.

## 5 Omics-informed nutritional modulation and bioactive compounds in MASLD

Bioactive compounds occupy an intermediate position between conventional lifestyle intervention and pharmacological therapy in MASLD. They should not be presented as disease-specific drugs or substitutes for weight loss, cardiometabolic risk control or approved pharmacological treatment. Rather, they may be considered diet-derived, food-associated or microbiota-derived modulators of the metabolic-inflammatory network underlying MASLD. From a multi-omics perspective, their relevance lies less in isolated biochemical effects but in whether their biological responses can be traced through lipidomic, metabolomic, proteomic and microbiome-related signatures.

### 5.1 Definition of bioactive compounds

In MASLD, bioactive compounds should be defined more broadly than dietary supplements. A practical framework is to regard them as diet-derived, food-associated or microbiota-derived factors capable of modulating host biological processes relevant to metabolic liver disease. This category includes plant-derived polyphenols, flavonoids, alkaloids and terpenoids, dietary lipids such as omega-3 polyunsaturated fatty acids, live microbial interventions such as probiotics, and gut microbiota-derived metabolites such as short-chain fatty acids and phenolic catabolites (121–124). For probiotics, the accepted definition remains live microorganisms that, when administered in adequate amounts, confer a health benefit on the host (121, 122). This broad definition is useful because bioactive compounds are highly heterogeneous in chemical structure, bioavailability, microbial metabolism and host exposure. Their effects may depend on baseline metabolic phenotype, gut microbiome composition, genetic background and concurrent dietary pattern. Therefore, in MASLD they are best viewed as network-level nutritional modulators rather than single-target therapeutic agents.

### 5.2 Mechanistic framework and evidence hierarchy

Bioactive compounds may influence MASLD through several convergent pathways, including AMPK, PPAR, SIRT1 and NF-*κ*B signalling. AMPK activation suppresses *de novo* lipogenesis and promotes fatty acid oxidation, whereas PPAR signalling regulates hepatic lipid handling and inflammatory tone. SIRT1 links nutrient sensing to mitochondrial function and stress responses, while NF-*κ*B connects lipotoxic stress with cytokine production and immune-cell recruitment. However, mechanistic plausibility should not be equated with clinical efficacy, because the level of evidence differs substantially across compounds. Among available examples, berberine provides relatively stronger human intervention evidence. In a randomized parallel-controlled trial of patients with MASLD, berberine added to lifestyle intervention reduced liver fat content more than lifestyle intervention alone and improved body weight, insulin resistance and lipid profiles (125). Experimental evidence further supports AMPK-dependent suppression of the SREBP-1c-SCD1 lipogenic programme as a plausible mechanism by which berberine attenuates hepatic steatosis (126). Nevertheless, even for berberine, evidence for histological improvement, fibrosis regression and long-term liver-related outcomes remains insufficient. Therefore, bioactive compounds should be discussed as biologically plausible modulators whose clinical relevance requires stronger human validation and molecular confirmation.

### 5.3 Multi-omics evidence for bioactive effects

Compared with conventional endpoints such as aminotransferases, ultrasound grading or liver fat content alone, multi-omics approaches provide a more informative framework for evaluating bioactive compounds in MASLD. They can reveal whether a nutritional intervention alters disease-relevant networks, including lipid composition, inflammatory proteins, bile acid signalling, microbial metabolism and host-microbiome interactions. This is particularly important because bioactive compounds often exert modest and pleiotropic effects that may be missed or oversimplified by traditional clinical markers. Omega-3 polyunsaturated fatty acids provide one of the clearer examples of human intervention-based omics evidence. In a plasma proteomic analysis from the randomized WELCOME trial, docosahexaenoic acid (DHA) plus eicosapentaenoic acid (EPA) supplementation altered proteins involved in coagulation, immune and inflammatory responses and lipid metabolism, including reductions in prothrombin and apolipoprotein B-100 (127). Lipidomic data further suggest that the response to omega-3 supplementation involves remodelling of circulating lipid species rather than a uniform reduction in hepatic fat. In patients with metabolic syndrome and MASLD, 12 months of n-3 PUFA supplementation was associated with changes in the plasma lipidome, while liver fat reduction was most evident when weight loss accompanied supplementation (128). These findings indicate that omega-3 effects are better interpreted as network-level cardiometabolic and lipidomic remodelling than as a simple liver fat-lowering effect. Gut-liver axis studies provide a second layer of evidence. In a 24-week double-blind randomized trial in patients with MASLD, curcumin supplementation reduced hepatic fat content and improved several metabolic indices. Integrated 16S rRNA sequencing and bile acid metabolomics further showed shifts in gut microbiota composition, increased serum deoxycholic acid, enhanced TGR5 expression in peripheral blood mononuclear cells and higher circulating GLP-1 levels (129). These data suggest that some bioactive effects may be mediated through microbiota-dependent bile acid signalling rather than direct hepatocellular actions alone. Similarly, omics approaches suggest that the active mediator may sometimes be a microbial metabolite rather than the parent dietary compound. For example, the gut microbial flavonoid catabolite 4-hydroxyphenylacetic acid increased in portal blood after flavonoid exposure and was sufficient to reverse diet-induced hepatic steatosis in mice (123).

### 5.4 Implications for precision nutrition

The broader implication of omics-based bioactive research is the development of precision nutrition in MASLD. Precision nutrition aims to integrate genetic susceptibility, metabolic phenotype, gut microbiome composition, dietary exposure and molecular response markers to identify who is more likely to benefit from a given dietary pattern or bioactive intervention. Metabolomics is particularly suited to this task because it can capture dietary exposure, bioactive compound levels and endogenous metabolic responses to nutritional interventions (130). This concept is especially relevant in MASLD because patients with similar liver fat content or fibrosis stage may differ substantially in insulin resistance, lipid handling, bile acid metabolism, genetic risk and microbial ecology. Evidence supporting gene-diet interaction in MASLD comes partly from the WELCOME trial, in which PNPLA3 genotype influenced changes in liver fat and tissue omega-3 incorporation during DHA plus EPA supplementation (127). Nutrigenetic analyses have also suggested that PNPLA3 risk allele carriers may show differential associations between macronutrient intake and hepatic steatosis or fibrosis risk (131). Microbiome-informed nutrition represents another emerging direction, as combined analyses of gut microbiome composition, genetic background and histological data have identified microbial taxa associated with hepatic injury features, including Faecalibacterium, Bacteroides and Prevotella-related patterns (131). At present, however, precision nutrition in MASLD remains a research framework rather than a routine clinical tool. Most available studies are observational, exploratory or based on small intervention cohorts, and prospective trials that allocate dietary or bioactive interventions according to genotype, microbiome profile or metabolic phenotype remain scarce. Current MASLD guidelines still emphasize weight loss, improved diet quality, reduced intake of ultra-processed foods and sugar-sweetened beverages, and Mediterranean-style dietary patterns as the foundation of care (18, 132). Therefore, omics-informed precision nutrition should be positioned as a future extension of standard lifestyle therapy rather than as a replacement for established dietary recommendations.

## 6 Clinical translation

The translational maturity of omics-derived findings varies substantially. Disease-associated molecular signatures should not automatically be regarded as clinically validated biomarkers. In this section, the term biomarker is used primarily for measurable molecular features or composite panels evaluated for a defined clinical purpose, whereas broader disease-associated molecular patterns are referred to as signatures. Therapeutic target discovery is also distinguished from biomarkers intended for treatment selection or response monitoring. For clinical translation, omics-derived biomarkers can be considered according to their intended context of use: disease detection, identification of at-risk MASH, fibrosis assessment, prognosis and risk stratification, treatment selection, and treatment-response monitoring. These applications should be distinguished because the level of evidence and the requirements for clinical validation differ substantially across contexts of use. The intended clinical applications, key supporting evidence and current maturity of representative omics-derived signatures and biomarkers are summarized in Table 2.

**Table 2:** Representative omics-derived signatures and biomarkers in MASLD according to clinical application and evidence maturity.

Omics domain	Representative biomarker/signature	Clinical purpose	Key supporting evidence/performance	Evidence maturity
RNA/protein multi-analyte panel	NIS2+	Detection of at-risk MASH	Serum-based model. Derivation: *n* = 198 (GOLDEN-505); validation: *n* = 684 and test: *n* = 2,035 (RESOLVE-IT). Test AUROC: 0.813 (95% CI 0.795–0.832), versus 0.653 for FIB-4 and 0.699 for ALT (both *p* < 0.0001) (15). A retrospective RESOLVE-IT screening simulation (*n* = 1,929) estimated biopsy screen-failure rates of 39% with NIS2 + at the cost-optimized cutoff of 0.53, versus 60% with the original screening pathway (134)	Advanced validation/clinically oriented
Lipidomics/metabolomics	MASEF	Detection of at-risk MASH; second-line assessment after FIB-4	Serum; international biopsy cohorts. Derivation: *n* = 790; external validation: *n* = 565. AUROCs: 0.76 (95% CI 0.72–0.79) and 0.79 (0.75–0.83). FAST AUROC was 0.74 in 311 validation participants; the reported AUROC comparison was not significant (*p* = 0.064). Sequential FIB-4→MASEF and FIB-4→VCTE-LSM pathways showed no significant difference in overall accuracy in 310 validation participants with available data (*p* = 0.69) (16).	External validation
Proteo-transcriptomics	ADAMTSL2/AKR1B10/CFHR4/TREM2 + BMI/T2DM model	Detection of histologically defined at-risk MASH/NASH (NAS ≥4 and F ≥ 2)	European patients with biopsy-proven NAFLD. Discovery: plasma, *n* = 191; paired liver RNA-seq was available in 52 cases. Independent validation: serum, *n* = 115. The four-protein plus clinical-variable model achieved AUROCs of 0.878 and 0.80, respectively. It outperformed FIB-4, NFS and AST/ALT in both cohorts. FAST comparisons were limited to subsets of 62 and 28 patients (50).	External validation
Metabolomics	Serum fibrosis and inflammation panels	Significant fibrosis (≥F2) and inflammatory activity	Serum; three same-center Chinese cohorts (total *n* = 293): discovery, *n* = 87 (63 biopsy-proven MASLD cases plus 24 controls); validation 1, *n* = 85 (biopsy); validation 2, *n* = 121 (non-biopsy)ᶜ. Fibrosis AUROCs: 0.928, 0.829 and 0.806, respectively. In validation 1, comparator AUROCs were 0.625 for FIB-4 and 0.729 for LSM. Inflammation AUROCs: 0.894 (discovery) and 0.776 (validation 1); sensitivity at the discovery cutoff was 20% in validation 1 (119).	Multi-cohort external validation
Genomics	*PNPLA3* rs738409 + *TM6SF2* rs58542926–augmented fibrosis models	Advanced-fibrosis risk refinement in high-risk MASLD	Genotyping plus clinical NIT data; 637 adults with biopsy-proven MASLD at two Korean centers. Derivation: *n* = 399; external validation: *n* = 238. In the validation T2DM subgroup (*n* = 121), genotype addition increased AUROC by 0.053 for NFS (*p* = 0.001) and 0.058 for FIB-4 (*p* = 0.010). The Agile 3 + gain was not significant (0.016; *p* = 0.058). Decision-curve gains in the full validation cohort were modest (39).	Targeted clinical development/validation
Genomics	Partitioned polygenic risk scores	Biological subtyping and liver-vs.-cardiometabolic risk stratification	Genotypes and imaging/clinical phenotypes; European-ancestry UK Biobank GWAS (*n* = 36,394). Six novel loci were replicated in four independent cohorts (total *n* = 3,903); this was locus replication. Two partitioned PRSs indicated liver-focused and systemic cardiometabolic risk patterns. Cross-sectional and prospective outcome associations were assessed in UK Biobank participants outside the imaging discovery sample (24).	Cohort-level validation/translational development
Integrative multi-omics	WGS+proteomics+phosphoproteomics+lipidomics+metabolomics molecular subtypes	Molecular classification and mechanistic stratification	Discovery liver proteomics: 103 biopsy-proven NAFLD cases and 17 non-NAFLD controls; other omics profiled subsets of liver, serum and urine samples. Three molecular subtypes were identified. Hepatic LAMB1/FLNC/ERCC3 multiplex immunofluorescence achieved 92.98% classification accuracy and AUROC 0.991 in 57 internal samples. External validation in 92 biopsy-proven patients from three Chinese hospitals reproduced subtype-associated molecular and histological patterns (107).	Exploratory/subtype discovery
Single-cell/spatial multi-omics	Human MASLD spatial multi-omics atlas	Mechanistic stratification of fibrotic and inflammatory niches	Human liver tissue: 61 individuals (10 controls, 17 MASL and 34 MASH). Assay subsets comprised 29 samples for single-cell RNA-seq, 35 for spatial transcriptomics and 27 for spatial metabolomics. A fibrosis-associated spatial gene program was enriched in advanced MASH and suggested profibrotic crosstalk between central vein endothelial cells and hepatic stellate cells (60).	Discovery/mechanistic

Evidence maturity is descriptive and is intended to indicate the current stage of biomarker development rather than a formal grading system. “Advanced validation/clinically oriented” does not imply routine guideline-endorsed clinical use. “At-risk MASH” generally refers to histologically confirmed MASH with an activity score ≥4 and significant fibrosis (≥F2), although exact criteria vary across studies. Historical “NASH” terminology is retained when it forms part of the original study endpoint or test name.

ALT, alanine aminotransferase; APRI, AST-to-platelet ratio index; AUC/AUROC, area under the receiver operating characteristic curve; FIB-4, Fibrosis-4 index; LSM, liver stiffness measurement; MASEF, metabolomics-advanced steatohepatitis fibrosis score; MASH, metabolic dysfunction-associated steatohepatitis; NFS, NAFLD fibrosis score; PRS, polygenic risk score; T2DM, type 2 diabetes mellitus; VCTE, vibration-controlled transient elastography; WGS, whole-genome sequencing.

### 6.1 Non-invasive omics-derived biomarkers

Non-invasive multi-omics biomarkers represent one of the most clinically relevant routes for translating MASLD omics research into practice. Their near-term value is unlikely to lie in population-wide screening for simple steatosis, which can already be detected by imaging and conventional clinical tools. A more realistic role is second-line or third-line risk enrichment in metabolically high-risk individuals, particularly for identifying at-risk MASH, clinically significant fibrosis and treatment-relevant disease. For disease activity assessment, blood-based molecular panels aim to capture biological signals that conventional liver enzymes or simple fibrosis scores cannot resolve. NIS4 and its optimized derivative NIS2 + are clinically oriented blood-based examples. NIS4 combines miR-34a-5p, YKL-40, *α*2-macroglobulin and HbA1c. NIS2 + simplifies this approach by using miR-34a-5p and YKL-40, with sex correction for miR-34a-5p. These panels integrate miRNA, protein and metabolic information to identify patients with at-risk MASH (15, 133, 134). Lipidomic and metabolomic models, including OWLiver and MASEF, extend this concept by using circulating lipid-related signatures, alone or in combination with clinical variables, to classify MASH or treatment-relevant at-risk disease (16, 135). The clinical value of these emerging omics-derived biomarkers should be interpreted against established non-invasive tests rather than in isolation. Direct comparative studies provide useful context. In the prospective validation of NIS2+, the AUROC for detecting at-risk MASH was 0.813, compared with 0.653 for FIB-4 and 0.699 for ALT (15). Similarly, MASEF achieved an AUROC of 0.79 in the external validation cohort, compared with 0.74 for FAST. A sequential FIB-4–MASEF strategy showed overall performance similar to that of FIB-4 followed by VCTE-based liver stiffness measurement p=0.69 (16). These findings suggest that selected omics-derived tests may improve the detection of treatment-relevant disease or provide alternative second-line strategies within existing non-invasive pathways. However, their clinical value should be judged according to the intended context of use, and the available evidence does not support replacing established fibrosis-oriented non-invasive tests across all clinical settings. These models are best understood as tools for referral enrichment and treatment evaluation rather than as replacements for liver biopsy, elastography or established fibrosis-oriented non-invasive tests. Fibrosis staging remains the most mature clinical application of omics-based biomarkers. Proteomic panels such as TLM3, plasma multi-protein signatures, APASHA and MSI-F aim to reflect fibrogenesis, matrix remodelling, metabolic stress and liver-extrahepatic tissue crosstalk (77, 78, 136, 137). Metabolite-based panels may also help stratify significant fibrosis and inflammatory activity, whereas extracellular vesicle-derived signatures provide an exploratory liquid-biopsy approach for advanced fibrosis risk (119, 138). However, most omics-derived fibrosis models remain limited by selected study populations, heterogeneous histological or imaging endpoints, modest sample sizes, platform dependence, assay cost, unclear decision thresholds and insufficient external validation. Therapeutic monitoring is an attractive but still immature extension of non-invasive multi-omics biomarkers. Current evidence is strongest for circulating proteomics, particularly aptamer-based serum proteomic signatures that can capture histological components of MASLD/MASH and drug-induced molecular responses (75). *Post hoc* analyses of pegbelfermin and acetyl-CoA carboxylase inhibitor trials suggest that proteomic signatures may reflect pharmacodynamic effects and pathway modulation (139, 140). More recent semaglutide studies further showed that SomaSignal-derived MASH components and broader aptamer-based proteomic signatures changed with treatment and correlated with histological, biochemical and metabolic measures (141, 142). Nevertheless, these proteomic signatures remain research-use tools rather than validated individual-level surrogate endpoints for MASH resolution or fibrosis regression. Overall, omics-derived non-invasive biomarkers provide a promising bridge between molecular discovery and clinical translation in MASLD/MASH. Their strongest current value lies in risk enrichment, mechanistic interpretation, disease-severity stratification and trial-based molecular assessment. Future studies should determine whether these tools can improve referral pathways, treatment eligibility assessment, patient selection, longitudinal monitoring and cost-effective management beyond established serum- and imaging-based non-invasive tests.

### 6.2 Precision medicine for MASLD

Precision medicine in MASLD should begin from a realistic premise. MASLD is a heterogeneous metabolic syndrome rather than a single uniform liver disease. Precision care does not require whole-omics profiling for every patient (21, 143). A more feasible approach is to identify clinically meaningful subgroups by integrating accessible clinical phenotypes with selected genetic and circulating molecular information. Importantly, molecular profiling should complement rather than replace conventional clinical assessment, particularly when routine variables already provide useful disease classification or prognostic information. The therapeutic landscape of MASH has also changed with the introduction of disease-directed pharmacological therapies, including resmetirom and semaglutide for selected patients with noncirrhotic MASH and moderate-to-advanced fibrosis (144, 145). These developments provide a more concrete clinical context in which the potential value of omics-derived biomarkers can be assessed. Possible applications include refinement of treatment eligibility, identification of biologically relevant patient subgroups and longitudinal assessment of treatment-associated molecular responses (146). However, evidence supporting the use of omics-derived biomarkers for individual treatment selection, treatment discontinuation or switching remains limited (146). Most currently reported treatment-associated molecular signatures should therefore be interpreted as exploratory or pharmacodynamic/response-associated signals rather than established predictive biomarkers.

Clinical and genetic stratification provide the first step toward this framework. Genetic evidence from partitioned polygenic risk scores supports the existence of biologically distinct MASLD patterns, including liver-predominant and systemic cardiometabolic forms (24). A similar concept has emerged from data-driven clinical clustering. Raverdy et al. used routine variables, including age, BMI, HbA1c, ALT, LDL cholesterol and triglycerides, to identify liver-specific and cardiometabolic MASLD subtypes with similar baseline liver severity but divergent clinical trajectories (22). This clustering strategy was further validated in large Asian cohorts. In these cohorts, the cardiometabolic subtype showed the highest risk of major adverse cardiovascular events, whereas the liver-specific subtype carried the highest risk of liver-related events (23). These findings suggest that MASLD stratification may be clinically useful even when based on accessible clinical variables rather than high-cost omics platforms. Genetic stratification is also moving from risk explanation toward clinical refinement. PNPLA3 and TM6SF2 are among the best-established genetic modifiers of MASLD progression. Notably, in patients with MASLD and type 2 diabetes, incorporating PNPLA3 rs738409 and TM6SF2 rs58542926 genotypes into established non-invasive fibrosis models produced modest incremental improvements in discrimination. The AUROC increased from 0.806 to 0.859 for NFS (*Δ*AUROC = 0.053) and from 0.793 to 0.851 for FIB-4 (*Δ*AUROC = 0.058), whereas the improvement for Agile 3 + was smaller (*Δ*AUROC = 0.016) (39). In another study, PNPLA3 risk allele carriage was associated with greater ALT reduction after semaglutide treatment, raising the possibility that genotype may influence biochemical response to therapy (147). However, these observations do not yet justify routine genotyping for all patients with MASLD. Their current value is more likely to lie in selected high-risk groups, trial enrichment, and hypothesis generation for pharmacogenomic studies. A more advanced form of precision medicine is to convert genetic risk into a therapeutic target. Fabbrini et al. reported phase 1 data showing that a GalNAc-conjugated PNPLA3 siRNA reduced liver fat in homozygous patients for the PNPLA3 I148M variant (148). Similarly, Mak et al. showed that ARO-HSD, a hepatocyte-targeted siRNA against HSD17B13, markedly reduced hepatic HSD17B13 expression and was accompanied by ALT reduction in early-phase studies (149). These studies illustrate how genetic and molecular evidence can move beyond disease classification to support therapeutic target discovery and genetically informed drug development. However, identification of a therapeutic target should not be equated with validation of a predictive biomarker for individual treatment selection. Histological efficacy, long-term safety, and outcome benefits still require confirmation. Proteomic stratification may provide a more clinically scalable route. Plasma and liver-secreted protein panels such as APASHA illustrate how circulating proteins can be used to stratify patients at risk of MASH (78). More recently, Xu et al. linked serum proteomic signatures with liver transcriptomic alterations across multiple cohorts, identifying proteins such as APOF, GSN, and LUM that were associated with MASLD severity and fibrosis-related molecular pathways (150). These studies suggest that plasma proteomics may help bridge peripheral blood signals and intrahepatic disease biology. However, clinical implementation will require simplified assays, external validation, cost-effectiveness analysis, and proof that proteome-guided decisions improve outcomes. Overall, omics-based precision medicine in MASLD is progressing from broad disease labeling toward biologically informed stratification. Its near-term value is more likely to lie in risk enrichment, trial design, biologically informed patient stratification and evaluation of treatment-associated molecular responses. It should not yet be interpreted as a ready-to-use replacement for standard clinical assessment or as a validated framework for individual treatment selection.

## 7 Discussion

### 7.1 Limitations of single-omics studies

Single-omics studies have greatly advanced the molecular characterization of MASLD, but their interpretative scope remains limited. Single-omics analyses are inherently limited by incomplete biological coverage, as each platform captures only one molecular layer and therefore cannot fully resolve the cross-layer interactions among genes, proteins, metabolites and cellular phenotypes that shape MASLD pathogenesis (151, 152). Second, limited reproducibility remains a major challenge. Omics data are highly sensitive to pre-analytical variables, assay platforms, normalization methods and batch effects. In MASLD, this problem is further amplified by differences in obesity, type 2 diabetes, alcohol exposure, ethnicity and disease stage across cohorts. Therefore, molecular signatures identified in one cohort require external validation and standardized analytical pipelines before they can be reliably generalized (153). Finally, single-omics studies also have limited causal resolution. Most are cross-sectional and observational, allowing associations with steatosis, inflammation or fibrosis to be identified, but not whether these signals are causal drivers or the consequences of disease. Genomic data can reduce reverse-causation bias and support risk stratification, but they do not capture dynamic disease activity or treatment response. Conversely, transcriptomic, proteomic and metabolomic signals are more disease-proximal, yet remain vulnerable to confounding. These limitations highlight the need for longitudinal and integrative multi-omics studies.

### 7.2 Challenges in multi-omics integration

Multi-omics integration remains technically demanding. Different omics layers vary in scale, distribution, missingness and technical noise, requiring careful preprocessing, normalization and batch correction before joint analysis (153–155). In MASLD, these challenges are further amplified by cohort heterogeneity, including differences in obesity, type 2 diabetes, alcohol exposure, ethnicity, medication use and fibrosis stage (18). High-dimensional datasets with limited sample size also increase the risk of overfitting and unstable feature selection. In addition, the cost of generating matched multi-omics data limits large-scale and longitudinal studies. Therefore, future work should prioritize harmonized protocols, transparent analytical pipelines, external validation and clinically interpretable models.

## 8 Conclusion

MASLD is a heterogeneous systemic metabolic disorder driven by multilayer biological regulation rather than a uniform liver-limited disease. Multi-omics technologies have expanded MASLD research from single biomarkers toward integrated molecular signatures that capture genetic susceptibility, transcriptional reprogramming, immune activation, fibrogenesis, lipid remodelling, metabolic dysfunction and gut-liver axis disturbance. These approaches have improved mechanistic understanding and generated promising non-invasive biomarkers for at-risk MASH, fibrosis stratification and treatment-response assessment. However, the field remains in transition. Most omics-derived signatures still require external validation, assay standardization, longitudinal confirmation and evidence of clinical utility beyond established non-invasive tools. Bioactive compounds, precision nutrition and precision medicine represent important translational extensions of MASLD multi-omics research, but their routine clinical application remains premature. Future progress will depend on integrating multi-omics with artificial intelligence, longitudinal phenotyping and prospective clinical trials to develop reproducible, interpretable and actionable tools for personalized MASLD management.
